# A New Fusion Fault Diagnosis Method for Fiber Optic Gyroscopes

**DOI:** 10.3390/s22082877

**Published:** 2022-04-08

**Authors:** Wanpeng Zhang, Dailin Zhang, Peng Zhang, Lei Han

**Affiliations:** School of Mechanical Science and Engineering, Huazhong University of Science and Technology, Wuhan 430074, China; m202070578@hust.edu.cn (W.Z.); m202170784@hust.edu.cn (P.Z.); m201970599@hust.edu.cn (L.H.)

**Keywords:** fiber optic gyroscope, fault diagnosis, SAE, WPD

## Abstract

The fiber optic gyroscope (FOG) is a high precision inertial navigation device, and it is necessary to ensure its reliability for effective use. However, the extracted fault features are easily distorted due to the interference of vibrations when the FOG is in operation. In order to minimize the influence of vibrations to the greatest extent, a fusion diagnosis method was proposed in this paper. It extracted features from fault data with Fast Fourier Transform (FFT) and wavelet packet decomposition (WPD), and built a strong diagnostic classifier with a sparse auto encoder (SAE) and a neural network (NN). Then, a fusion neural network model was established based on the diagnostic output probabilities of the two primary classifiers, which improved the diagnostic accuracy and the anti-vibration capability. Then, five fault types of the FOG under random vibration conditions were established. Fault data sets were collected and generated for experimental comparison with other methods. The results showed that the proposed fusion fault diagnosis method could perform effective and robust fault diagnosis for the FOG under vibration conditions with a high diagnostic accuracy.

## 1. Introduction

**Figure 1:**
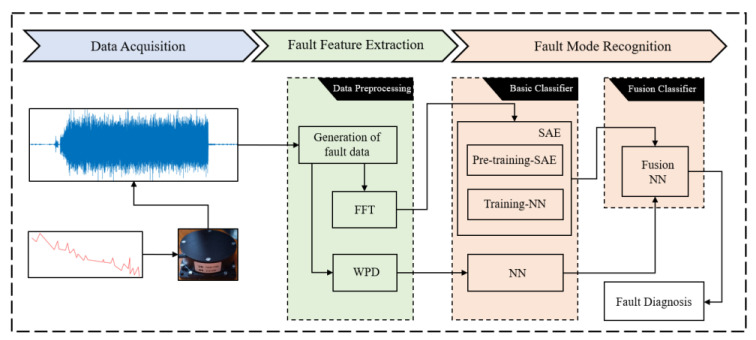
The framework of the proposed fusion model to FOG fault diagnosis.

Fault diagnosis is aimed to identify the type, location, time, and size of a fault [1]. In this paper, fault diagnosis of FOG is defined as the ability to effectively identify the normal state of an FOG, as well as five different types of faults. With the development of machine learning, data-driven fault diagnosis methods are widely used. In these methods, the historical data of sensors becomes the main means of fault identification. The process of diagnosis can usually be divided into three steps: signal processing, feature extraction, and fault identification, as shown in Figure 1. The original data are converted into low-dimensional features with better classification performance. Then, the features are fed into the classifier to achieve the identification and classification of faults. Now, more and more intelligent algorithms have been used for fault diagnosis of industrial equipment, the integration of which has greatly improved the accuracy of diagnosis and ensures the stable operation of equipment under actual working conditions [2]. Neural networks [3,4], support vector machines [5], and random forests [6,7] are widely used for the fault diagnosis of rolling bearings. Zahoor et al. [8] proposed the information ratio principal component analysis (Ir-PCA) method, which is applied to the fault diagnosis of multistage centrifugal pumps (MCP); Nguyen et al. [9] proposed a Stacked sparse autoencoder-based deep neural network (SSA-DNN) for the diagnosis of variable speed gear faults. In more complex industrial scenarios, methods such as transfer learning [10,11], ensemble learning [12,13] wavelet packet transform [14,15,16], and clustering [17,18] are also widely adopted. These intelligent algorithms have played a significant role in the fault diagnosis of modern industrial equipment and sensors.

The fiber optic gyroscope is an angular velocity-sensitive detection device with high measurement accuracy, which can even be used for a long time under harsh working conditions. FOG plays a complementary role for laser targeting in shield attitude measurement systems [19,20]. Therefore, it is essential to diagnose the faults of FOG and obtain its fault classes so as to ensure the stability and reliability of the sensors during construction. Yu et al. [21] proposed the genetic algorithm based on a cloud-model and radial basis function neural network for the online fault diagnosis of gyroscopes; Liu et al. [22] presented the least squares support vector machine based on wavelet packet decomposition for gyroscope fault diagnosis. In terms of FOG fault diagnosis, Chen et al. [23] put forward an improved sparrow search algorithm based on the support vector machine method, while Guan [24] proposed a CNN-based deep feed forward network.

Vibrations generated by the running shield will cause the measurement of FOG to be out of calibration or invalid [25]. Although many intelligent methods have obtained exciting results in FOG fault diagnosis, most of them collect or generate fault data under ideal conditions, and do not take into account the influence of vibration. Signal interference caused by random vibrations of various frequencies during construction [26,27] will combine the fault signal features of the fiber optic gyroscope with the vibration signal features, further reducing the extraction ability of the fault features, thereby decreasing the diagnosis accuracy [28]. To solve this problem, a new fusion fault diagnosis method for FOG is proposed in this paper, according to the outstanding contribution of neural networks in fault diagnosis. Firstly, two feature extraction methods, FFT and WPD, are adopted in this paper to extract fault features. Secondly, two classifiers with high diagnostic accuracy, FFT-based SAE and WPD-based NN, are established. Finally, a fusion fault diagnosis network is constructed with the output of the basic classifier as the feature vector. The fusion model proposed is compared with other fault diagnosis methods, including BP, SVM, CNN, and random forest (RF), etc., to verify the effectiveness of the fusion model. The contributions of this paper can be summarized as follows:

(1). In this paper, five failure modes of FOG are established. The complementary characteristics of the frequency domain features and energy statistical features of the fault data under vibration conditions are verified by normalized Wasserstein distance.

(2). In this paper, a fusion model is proposed for FOG fault diagnosis. The model combines two neural network diagnostic classifiers of different depths. This approach can improve the accuracy and robustness of fault detection under vibration conditions, compared to existing fault diagnosis methods.

## 2. Materials and Methods

The framework of the fusion model proposed is shown in Figure 1. The data of FOG under vibration conditions were collected in the data preprocessing stage to generate fault data. The second stage is the training of two basic classifiers, namely a sparse auto encoder neural network based on fast Fourier transform, and a neural network based on wavelet packet decomposition. The third stage is the training of the fusion classifier. In this section, the theoretical basis and parameter settings of these three classifiers will be further explained.

### 2.1. Sparse Auto Encoder Neural Network Based on Fast Fourier Transform

**Figure 2:**
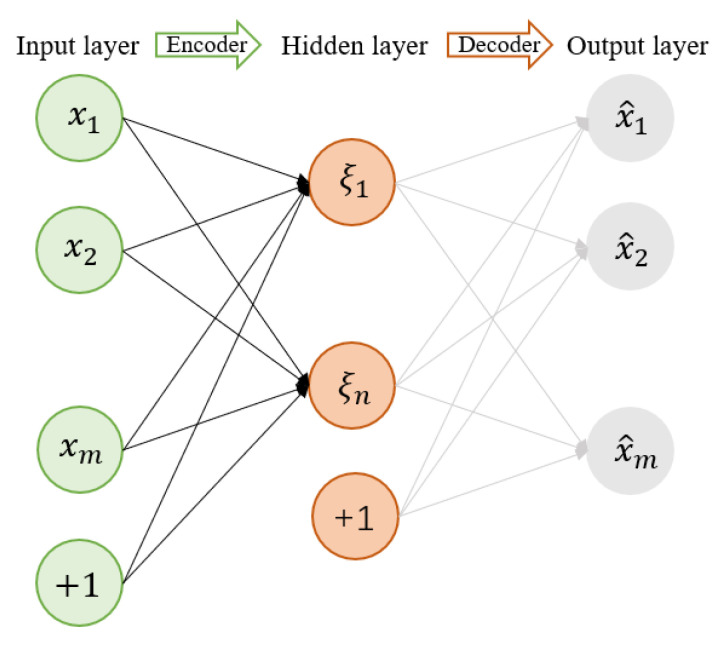
The schematic of the auto encoder.

The auto encoder is a system that reduces the dimensionality of its input. The auto encoder consists of an encoder and a decoder part, as shown in Figure 2. The main purpose of the auto encoder is to transform the input x from a high-dimensional space into a low-dimensional feature space to obtain the intermediate variable *ξ*. Then, ξ is reconstructed from the feature space into an $\hat{x}$. Then the difference between x and $\hat{x}$ is minimized. When the dimensionality of the intermediate variable ξ (the node numbers in the hidden layer) is smaller than the dimensionality of the input x (the node numbers in the input layer), the intermediate variable ξ can be regarded as the dimensionality reduction data, compressed with minimal information loss to the input data. Since no label information is utilized in the encoding and decoding process, it can be used in an unsupervised task. Many variants of the auto encoder, such as the denoising auto encoder [29], shrinking AE [30], and SAE [31,32], are created to better learn feature reformulation and avoid overfitting. A sparse auto encoder (SAE) is actually a sparse constraint added to the encoding to penalize the activation output values of the hidden layer [33]. In an SAE, the hidden cell $\rho_{k}(k=1,2,\cdots n)$ can be calculated by Equation (1), where $f_{s}$ is the activation function of Sigmoid and it will be close to zero, which means that most of the hidden layer is suppressed. The Kullback–Leibler (KL) divergence for measuring these deviations can be written as Equation (2):

(1) $$\rho_{k}=\frac{1}{n}\sum_{i=1}^{n}\left[f_{s}\left(b_{k}+W_{ik}x_{i}\right)\right]$$

(2) $$\mathrm{KL}\left(\rho ∥\rho_{k}\right)=\rho \log\frac{\rho }{\rho_{k}}+(1-\rho )\log\frac{1-\rho }{1-\rho_{k}}$$

In addition, the loss function needs to be regularized to prevent overfitting. β and γ are the penalty coefficients of the sparse and regular terms, respectively. The total cost function of SAE in pre-training is shown as Equation (3), where $\sum_{i=1}^{n}L\left(x_{i},{\hat{x}}_{i}\right)$ is the mean square error (MSE) loss of decoding ${\hat{x}}_{i}$ and the true $x_{i}$

(3) $$J_{\mathrm{SAE}}(\theta )=\sum_{i=1}^{n}L\left(x_{i},{\hat{x}}_{i}\right)+\beta \mathrm{KL}\left(\rho ∥\rho_{k}\right)+\frac{\gamma }{2}\sum_{i=1}^{m}\sum_{k=1}^{s}\left(W_{ik}^{2}+W_{ki}^{\prime 2}\right)$$

**Figure 3:**
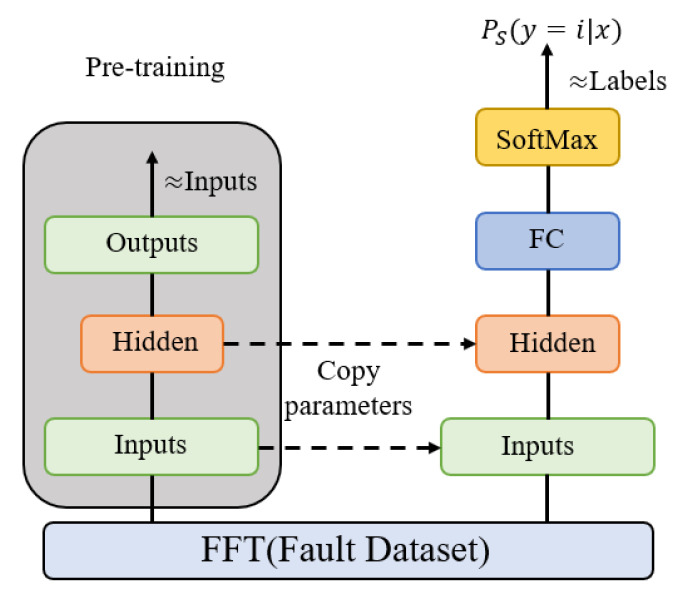
The sparse auto encoder neural network is based on FFT.

After pre-training, the parameters of the input and hidden layers are copied to the classifier, and the total loss function of the classifier is given by the output of the fully connected and SoftMax layers, as shown in Figure 3. In addition, the cross-entropy loss function is shown as:

(4) $$L=\frac{1}{N}\sum_{i}L_{i}=-\frac{1}{N}\sum_{i}\sum_{c=1}^{M}y_{ic}\log\left(p_{ic}\right)$$

where *M* is the number of categories, $y_{ic}$ is the sign function 0 or 1, *N* is the number of samples, and $p_{ic}$ is the predicted probability that observation sample i belongs to class c.

The input data with FFT will still be combined with fault features and vibration frequencies. At this time, the sparse auto encoder can obtain better low-dimensional features, and further improve the diagnostic accuracy.

### 2.2. Neural Networks Based on Wavelet Packet Decomposition

Wavelet packet decomposition (WPD) can extract the features of the full-band information of the FOG signal under vibration conditions.

**Figure 4:**
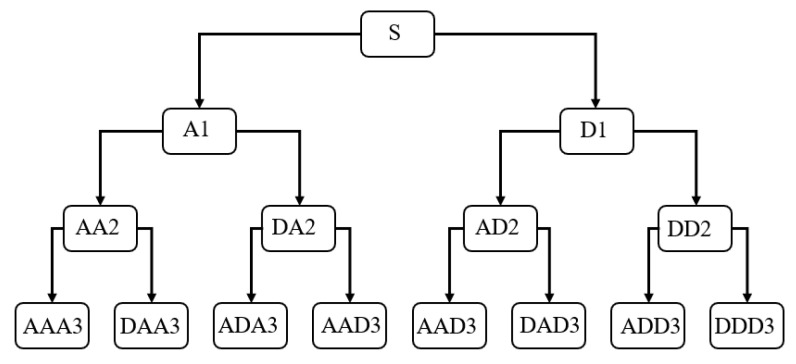
The schematic of wavelet packet decomposition.

Figure 4 shows the schematic of the three-layer wavelet decomposition. In the figure, A represents the approximation filtering, which can extract the low frequency part of the previous decomposition; D represents the detailed filtering, which can extract the high frequency part of the previous decomposition. The frequency and intensity of the vibration received by the FOG are different due to the different degrees of abrupt change at fault. Thus, the db10 wavelet of the Daubechies wavelet family with good symmetry and regularity is chosen to carry out a seven-layer decomposition. It can better extract the characteristics of the FOG output signal in each frequency band.

For a given set of orthogonal scale transform functions and wavelet functions, the following conditions should be satisfied:

(5) $$\{\begin{array}{l} \phi (t)=\sqrt{2}\sum_{k}h_{k}\phi (2t-k)\\ \psi (t)=\sqrt{2}\sum_{g}g_{k}\phi (2t-k) \end{array}\;k\in Z$$

ϕ(t) is the orthogonal scale transform function; ψ(t) is the wavelet function; $h_{k}$ is the orthogonal conjugate filter of ϕ(t); $g_{k}$ is the orthogonal conjugate filter of ψ(t). Make $u_{0}(t)=\phi (t),u_{1}(t)=\psi (t)$:

(6) $$\{\begin{array}{l} u_{2n}(t)=\sqrt{2}\sum_{k}h_{k}u_{n}(2t-k)\\ u_{2n+1}(t)=\sqrt{2}\sum_{g}g_{k}u_{n}(2t-k) \end{array}$$

$u_{n}$ is the wavelet packet function, and the corresponding wavelet packet decomposition algorithm is:

(7) $$\{\begin{array}{l} d_{j}^{2n}[k]=\sum_{l\in Z}h_{l-2k}d_{j+1}^{n}[l]\\ d_{j}^{2n+1}[k]=\sum_{l\in Z}g_{l-2k}d_{j+1}^{n}[l] \end{array}$$

$d_{j}^{2n}$ is stranded wavelet packet reconstruction coefficients, which are level j, and number 2n. Calculating the energy of each frequency band of the signal after wavelet packet decomposition, according to Parseval theorem, the energy of the signal is expressed as:

(8) $$∥x(t){∥}^{2}=\int_{-\infty }^{\infty }|x(t){|}^{2}dt$$

In the wavelet packet decomposition, the following relationship exists:

(9) $$\int_{-\infty }^{\infty }|x(t){|}^{2}dt=\sum_{j}\sum_{k}{\left|d_{j}^{k}\right|}^{2}$$

$d_{j}^{k}$ is wavelet packet reconstruction coefficients, as in Equation (7). Therefore, we can calculate the signal energy of each layer in the wavelet packet decomposition process through the wavelet packet reconstruction coefficients.

**Figure 5:**
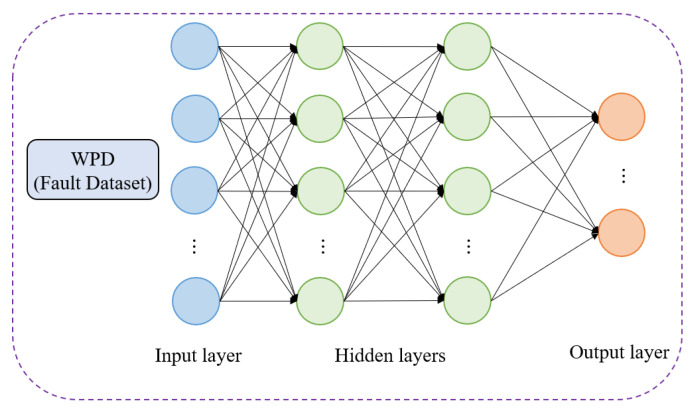
The neural network based on WPD.

After the decomposition is completed, the decomposed energy characteristics are inputted to the input layer of the classifier, and the total loss function of the classifier is as in Equation (4) via the fully connected layer and the SoftMax layer, as shown in Figure 5.

### 2.3. Fusion Fault Diagnosis Model

In the vibration condition, the fault feature extraction ability and generalization of a single model are relatively weak. In this section, the advantages of the above two classifiers are integrated through model fusion to improve the prediction accuracy of the model. The voting method, a commonly used ensemble learning method, generally integrates the labeling results of multiple basic classifiers to select the one with the most output. However, individual classifiers have different detection capabilities for each fault class. Moreover, it is difficult to ensure the stability of the fusion effect when the number of base classifiers is small. For these reasons, the stacking method [34] was chosen for model fusion in this section. Stacking is a multilayer model in which two trained models are used as base classifiers, and their predicted probability results are used as a new training dataset to create a new learner that integrates the results of the two basic classifiers. The first layer of learners is defined as the basic classifier, and the second layer of learner is called the fusion classifier.

**Figure 6:**
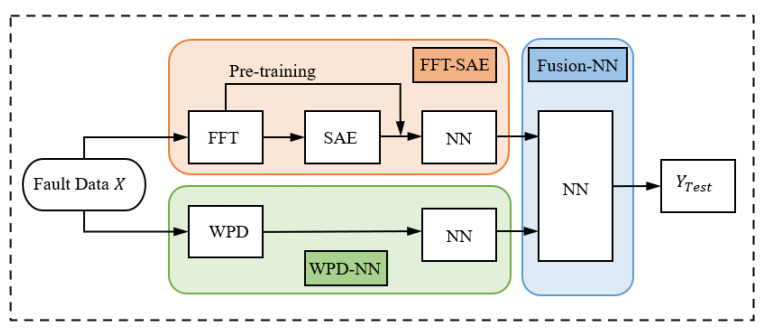
The schematic of the fusion model.

As shown in Figure 6, the two basic classifiers in this paper are FFT-based SAE and WPD-based NN. We divided the training data into basic training data and fusion training data. Firstly, the two base classifiers were trained with the basic training data. After the training was completed, the fusion training data were spliced by the 6-class output probabilities of the base classifiers to obtain the 12-dimensional data for fusion training. The fusion classifier was trained with these data to obtain the complete fusion model parameters. The parameters of each of these classifiers are listed in the Table 1.

## 3. Results

In this section, FOG vibration experiments were designed to generate fault data, and fault diagnosis experiments were conducted to verify the feasibility of the proposed method. The FOG used in the experiment is a single-axis interferometric closed-loop FOG, FOGS170A, and the technical parameters are shown in Table 2. In total, 1000 sets of data were collected under each fault class to ensure the stability of the data.

### 3.1. Data Preparation

#### 3.1.1. Mathematical Model of FOG

**Figure 7:**
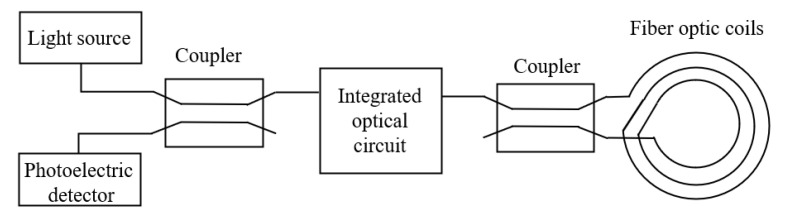
Physical model of FOG.

In order to establish the mathematical model of FOG, the physical model needs to be studied first. The subject of the interferometric FOG is a Sagnac interferometer, which consists of a light source, coupler, light detector, integrated optical circuit, and fiber optic coil, shown as Figure 7. The light from the light source is directed into the fiber coil from the opposite direction, and, due to the Sagnac effect, when the two beams are reunited at the origin, we can measure the differential phase shift, and calculate the velocity based on the interference effect produced by the two beams [24].

For the fiber optic gyroscope drift model [35], the effect can be described as:

(10) $$\omega (t)=\omega_{0}(t)+\varepsilon (t)$$

where ω(t) is the output angular velocity of FOG, $\omega_{0}(t)$ is the actual angular velocity of FOG, and ε(t) is the total drift of FOG.

(11) $$\varepsilon (t)=\varepsilon_{0}+A\sin\left(2\pi ft+\theta_{0}\right)+\sigma n(t)+w(t)$$

where ε_0_ is the constant drift of FOG, A is the amplitude of the periodic component, σn(t) is the white noise with intensity σ, and w(t) is the colored noise.

(12) $$w(t)=\frac{4n(k-1)\sin(n(k))}{1+n^{2}(k-1)}$$

where n(k) is the sequence of white noise.

#### 3.1.2. Generation of Fault Data

**Figure 8:**
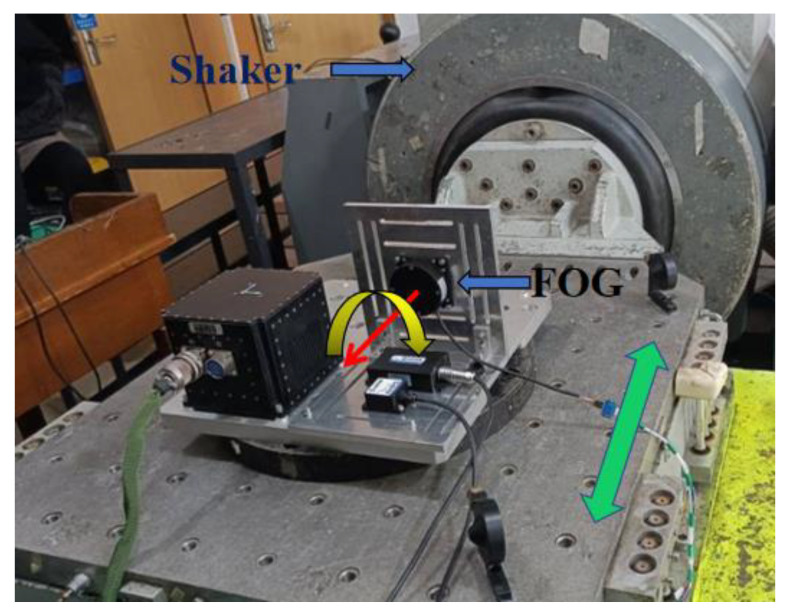
Experimental conditions of FOG. The green arrow represents the vibration direction of the shaker; the yellow arrow represents the positive direction of the angular velocity measured by the FOG.

**Figure 9:**
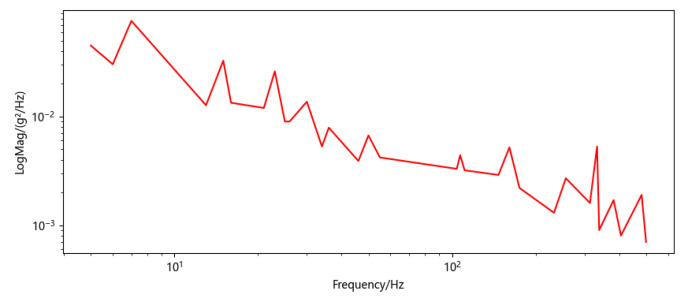
Random vibration power spectrum density.

**Figure 10:**
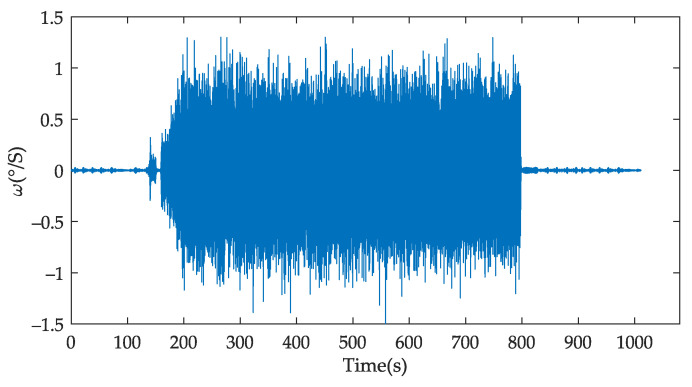
The output of FOG with the random vibration condition.

The experiment was arranged as shown in Figure 8, with the FOG placed on the shaker, and the shaker vibrating horizontally. The national military standard vibration test standard GJB150.16A-2009 Schedule C.7 vibration spectrum was used to simulate random vibration, and the frequency spectrum is shown in Figure 9. Frequency spectrum specific inflection point data can be found in Appendix A Table A1. The sampling frequency of the FOG was 100 HZ, vibration time was 600 s, and a total of 1080 s data was collected. The output curve of FOG angular velocity is shown in Figure 10.

Since it is very difficult to obtain a large amount of fault data for FOG, the mathematical simulation method was selected in this paper to generate the fault data [35,36,37]. The generation of fault data was carried out based on real data. Each sample took 10 s data, 1000 points, offset 1 s, and generated 1000 groups of samples for each class under six fault classes, such as normal data and bias fault, blocking fault, drift fault, cycle fault, multiplicative fault, with a total 6000 groups of data samples.

**Figure 11:**
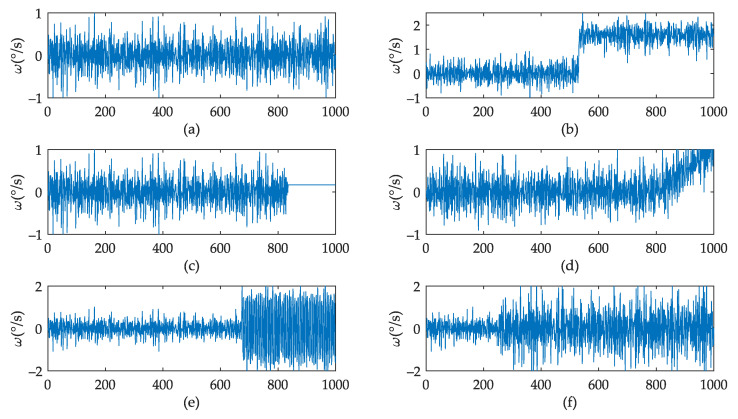
Fault classes of FOG. (**a**) normal data. (**b**) bias fault data. (**c**) blocking fault data. (**d**) drift fault data. (**e**) periodic interference fault. (**f**) multiplicative fault data.

Bias fault: FOG is subjected to a strong vibration shock. The fiber ring may be offset or deformed, resulting in a certain constant deviation of the output signal from the signal in its normal state. The mathematical model is shown in Equation (13), in which y is the output of the FOG in its normal state, x is the number of sampling points, and $x_{s}$ is the number of points in the range [100, 900] when the fault occurs. $k_{1}$ is the bias amount, and the random range is ±[0.02,0.2]. The generated fault data are shown in Figure 11b.

(13) $$y=\{\begin{array}{cc} y(x) & \;\;\;x<x_{s}\\ y(x)+k_{1} & \;\;\;x\geq x_{s} \end{array}$$

Blocking fault: This refers to a point at which the gyro signal transmission is affected from a certain point onwards, keeping the same output value from the previous moment. The mathematical model is shown in Equation (14). The generated fault data are shown in Figure 11c.

(14) $$y=\{\begin{array}{cc} y(x) & \;\;\;x<x_{s}\\ y(x_{s}) & \;\;\;x\geq x_{s} \end{array}$$

Drift fault: This is one of the most common types of failure in FOG, often due to changes in the working environment or internal parameters, such as temperature changes, causing the output of the FOG to have an increasing constant term. The output value tends to increase over time. The mathematical model is shown in Equation (15), where $k_{2}$ is the coefficient of drift, and random range is ±[0.002,0.01]. The generated fault data are shown in Figure 11d.

(15) $$y=\{\begin{array}{cc} y(x) & \;\;\;x<x_{s}\\ y(x)+k_{2}(x-x_{s}) & \;\;\;x\geq x_{s} \end{array}$$

Periodic interference fault: This means that the output signal of the FOG is attached to a periodically changing signal from a certain moment. The mathematical model is shown in Equation (16), where square(x) is a square wave with the same frequency as the gyroscope sampling frequency, and the amplitude random range is ±[0.02,1.60].The generated fault data are shown in Figure 11e.

(16) $$y=\{\begin{array}{cc} y(x) & \;\;\;x<x_{s}\\ y(x)+square(x-x_{s}) & \;\;\;x\geq x_{s} \end{array}$$

Multiplicative fault: The output signal of the FOG is multiplied by a constant from a certain point onwards due to a drastic change in the FOG scale factor, which is in turn caused by a drastic change in the operating environment. The mathematical model is shown in Equation (17), where $k_{3}$ is the multiplier factor, and random range is [2,8].The generated fault data are shown in Figure 11f.

(17) $$y_{s}=\{\begin{array}{cc} y(x) & \;\;\;x<x_{s}\\ k_{3}y(x) & \;\;\;x\geq x_{s} \end{array}$$

**Figure 12:**
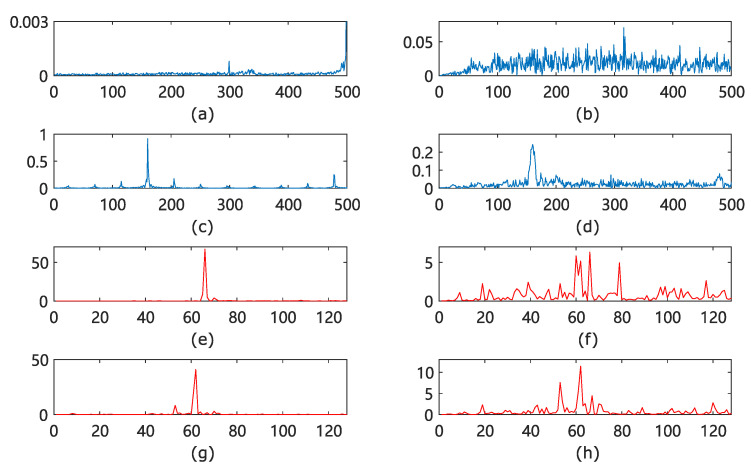
Preprocessing of fault data. (**a**) FFT of normal data. (**b**) FFT of normal data with vibration. (**c**) FFT of periodic interference fault. (**d**) FFT of periodic interference fault with vibration. (**e**) WPD of normal data. (**f**) WPD of normal data with vibration. (**g**) WPD of periodic interference fault. (**h**) WPD of periodic interference fault with vibration.

Figure 11 shows the time domain figure of the fault signal. In order to obtain better detection results, we need to preprocess the signal and extract more distinguishing features. Figure 12 shows the preprocessing effect of the FFT and WPD methods.

Fault diagnosis is a process in which the diagnostic model effectively distinguishes signals of different fault types, but the interference of vibration increases this difficulty. Figure 12a,b show the normal signal FFT processing in stationary and vibration states, respectively. Figure 12c,d show the fault signal (periodic disturbance fault) FFT processing in stationary and vibration states, respectively; Figure 12e–h show the WPD processing of the same condition with (a–d). From the figures, it can be seen that vibration is disturbing for the diagnosis of the fault classes.

To further illustrate the difference between the two processing methods, we adopt the Wasserstein distance to measure the difference of data distribution under different fault classes, and the larger Wasserstein distance indicates that the greater difference between the two classes, the more helpful to the diagnosis of fault classes.

**Figure 13:**
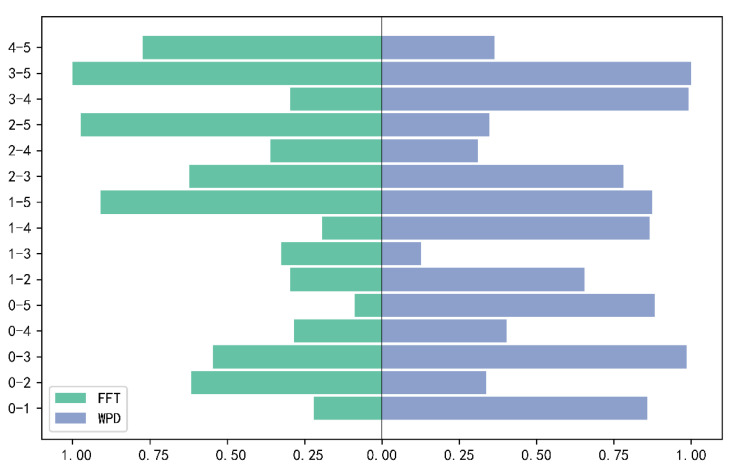
Normalized Wasserstein distance taper figure for two processing methods.

Figure 13 shows the normalized Wasserstein distance between different fault classes after FFT and WPD processing, with ‘0–1’ representing the Wasserstein distance between class 0 and class 1, a symmetric value with 1–0. After the two processing methods, ‘0–5’ and ‘1–3’ are the smallest, respectively. This is the same as the difference between two basic classifiers diagnosed in the subsequent experimental validation. The two processing methods have different focuses for the same two classes, which indicates, to a certain degree, that the two methods have the complementary possibilities.

### 3.2. Experimental Results

In this section, three experiments are designed. First is the parameter selection experiment of the number of training data for the basic classifier and fusion classifier. Secondly, the stability of the model is demonstrated by five sets of cross-validation experiments. Finally, the proposed fusion model is compared with other diagnostic methods. All models in this paper are built on the Pytorch platform, and all experiments are implemented on a computing platform configured with an NVIDIA GTX 1660Ti GPU and 16 GB of RAM. The experimental results are the average of five experiments.

#### 3.2.1. Parameter Selection Experiment

The fusion fault diagnosis method proposed in this paper is divided into two stages of classifier training. The first stage of training requires a large amount of data to ensure the performance of the basic classifier, and the second stage of training further reduces the number of input features, but still requires a sufficient amount of data to fit the distribution of test data. In this experiment, the fault dataset is divided into 80% labeled training data and 20% unlabeled test data, and the labeled data are divided into several groups with different proportions for two stages of training in order to find the most suitable proportion.

In Table 3, the ratio represents the ratio of the basic classifier and fusion classifier training data. It can be noticed from Table 3 that the accuracy of the two basic classifiers increases with the first layer training data from 93.19% and 91.94% to 95.50% and 94.44%. However, the fusion classifier is the highest at the ratio of 6:2, measuring 98.23%. It is understandable that, if the fusion classifier training data are excessive, the accuracy of the basic classifier is low, resulting in low overall accuracy; if the training data of the fusion classifier are insufficient, overfitting will reduce the accuracy, and increase the standard deviation, resulting in poor stability. Therefore, the following experiments are conducted based on a ratio of 6:2.

#### 3.2.2. Stability Experiments of the Fusion Model

Since the fault characteristics of vibration data and stationary data are quite different, in order to verify the stability of the model and avoid the interference of specific samples, this experiment uses the cross-validation method to validate the fusion model. Specifically, all samples are divided into five parts equally, one part is used as the training data, one part is used as the test data of the model, and the rest is the training data of the basic classifier.

**Figure 14:**
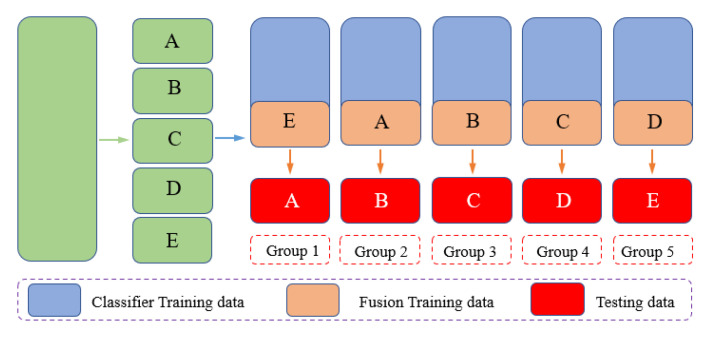
The diagram of cross-validation.

**Figure 15:**
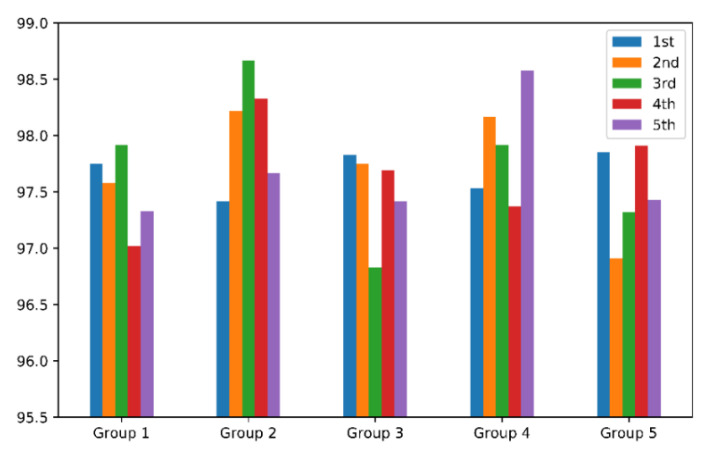
Bar chart of accuracy (%) with five groups cross-validation.

As shown in Figure 14, we divided the fault data into five equal parts in order, categorized as ‘A’–‘E’. A total of five groups were divided, and each group was selected different test samples for five experiments. The histogram was obtained, as shown in Figure 15.

In Figure 15, five experiments were conducted in each group, and the average accuracy and standard deviation of each group were 97.52 ± 0.35%; 97.73 ± 0.53%; 97.5 ± 0.40%; 97.91 ± 0.49%; and 97.48 ± 0.41%, respectively. The overall average accuracy was 97.63%, which was similar in each group; the standard deviation between groups was 0.19%, which was smaller than the standard deviation within each group. It indicates that the accuracy is little affected by the group and sample, and the model is relatively stable.

#### 3.2.3. Comparisons with Other Methods

In this section, two sets of experiments are compared in order to reflect the performance of the proposed fusion algorithm. (1) Two basic classifiers are used as comparisons to demonstrate that the proposed fusion model can further improve diagnosis accuracy based on the basic classifier, (2) as compared with BP, SVM, optimized SVM, CNN, and random forest, which are popularly used as fault diagnosis algorithms. It proves that the fusion model proposed has higher accuracy.

1. Comparison with basic classifier.

**Figure 16:**
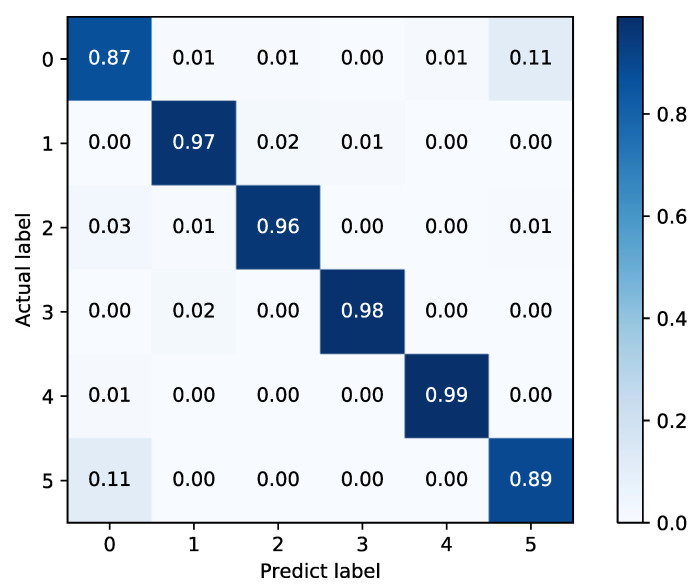
Confusion matrix of FFT + SAE.

**Figure 17:**
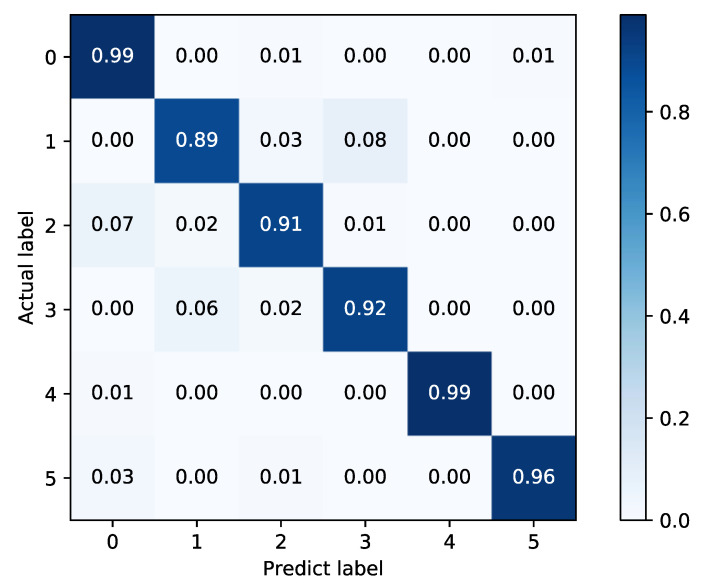
Confusion matrix of WPD + NN.

As shown in Table 4, the proposed fusion model has a 3.22% improvement over FFT + SAE, and 2.31% improvement over WPD + NN, which indicates that the fusion model can further improve the diagnosis accuracy. The confusion matrices of the two basic classifiers are shown in Figure 16 and Figure 17.

**Figure 18:**
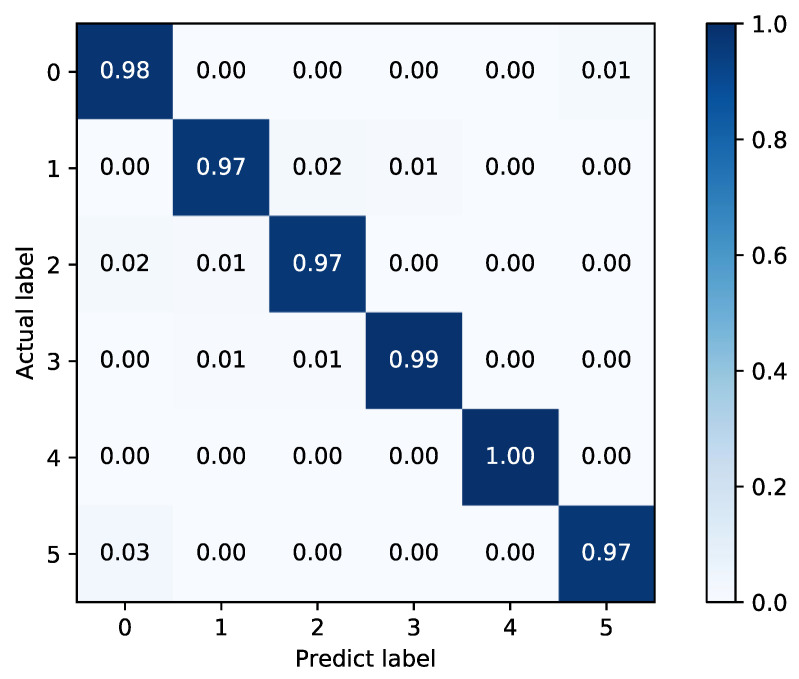
Confusion matrix of the fusion model.

In Figure 16 and Figure 17, it can be found that the FFT + SAE classifier has a larger misclassification rate in labels 0 and 5, while 1 and 3 are classified more accurately, which is exactly the opposite in the WPD + NN classifier. Therefore, the combination of these two complementary methods can be more effective for fault diagnosis, as shown in Figure 18.

2. Comparison with other methods.

To achieve a better comparison, we used the same test dataset (20%) for the proposed fusion model and other methods.

BP and SVM are commonly used intelligent algorithms in fault diagnosis. In this experiment, the Gaussian kernel function of SVM was used, and parameters C and gamma were set as default. BP was a three-layer network with node numbers 1000, 512, 64, and 6 used. The DFF network [24] extracted features through two layers of Conv, and the Conv 1 parameters were [4, 11, 2, 5, 2], representing the kernel number = 4, kernel size = 11, stride = 2, padding = 5, MaxPool = 2. The Conv 2 parameters were [8, 7, 1, 3, 2]. The node number of the FC layer was the same as that of BP. ISSVM [21] is an optimized SVM with an improved sparrow search algorithm. The C range was [1, 100], gamma ranges was [10–5, 0.1], optimization C = 57.75, and gamma = 0.0048. These two methods further improved the accuracy of diagnosis compared to the BP network and SVM methods, which proved the importance of feature extraction for the original signal. Further, the random forest (RF) method of integrated learning was considered, where the number of trees is 100, and each decision tree is an independent classifier. RF is a method that combines the diagnostic results of these trees. However, it only considers one model, namely the decision tree, therefore it is easy to over-fit, thereby leading to a lower accuracy. The fusion model proposed in this paper integrated these problems, adopted two different feature extraction methods, and selected the different network according to these feature vectors. The comparison results, shown in Table 5, revealed that the fusion model enjoys higher diagnosis accuracy than other methods.

## 4. Conclusions

In this paper, a fusion method was proposed for the fault diagnosis of FOG under vibration conditions. To solve inaccurate fault feature extraction caused by the vibration noise interference of FOG, the proposed method fuses a sparse auto encoder diagnostic model based on fast Fourier transform and a neural network diagnostic model based on wavelet packet decomposition. In the experiments, a total of 6000 sample fault datasets, obtained by simulating the output data under random vibration of FOG, were used for algorithm training and testing. The experimental results showed that the proposed method can diagnose the fault of FOG more accurately under vibration conditions compared with existing methods. Once the model is trained, it can be simply imported and reused in a short time. The time consumed for each step is shown in Appendix B Table A2. The method can be used in the shield construction environment, and significantly improve the reliability of the FOG measurement system.

## Figures and Tables

**Table 1:** Structure and hyperparameter settings of the three classifier models.

Model		Structure (Units and Activation)	Hyperparameter
FFT + SAE	Pre-training	Dense (1000, 128); activation = ‘Sigmoid’ Dense (128, 1000)	Max epochs = 10,000; Batchsize = 6000
			Beta = 0.01
			Optimizer = Adam (*lr* = 0.005)
	Training	Dense (128, 64); activation = ‘Relu’	Max epochs = 10,000; Batchsize = 3600
		Dense (64, 32); activation = ‘Relu’	Optimizer = Adam (*lr* = 0.005)
		Dense (32, 6); activation = ‘SoftMax’	Dropout rate 0.2
WPD + NN		Dense (128, 64); activation = ‘Relu’	Max epochs = 10,000; Batchsize = 3600
		Dense (64, 32); activation = ‘Relu’	Optimizer = Adam (*lr* = 0.005)
		Dense (32, 6); activation = ‘SoftMax’	Dropout rate 0.2
Fusion model		Dense (12, 6); activation = ‘SoftMax’	Max epochs = 10,000; Batchsize = 1200
			Optimizer = Adam (*lr* = 0.005, weight decay = 0.0001)

**Table 2:** Sensor technical parameters.

Gyroscope Type:	FOGS107A
Zero bias stability:	<0.05°/h(10s,1σ)
Zero bias repeatability:	<0.05°/h(10s,1σ)
Random wandering factor:	<0.005°/√h
Dynamic measurement range:	±500°/s
Output method:	RS422

**Table 3:** The accuracy (%) table with different parameters.

Ratio	WPD + NN	FFT + SAE	Fusion	std (%)
4:4	93.19	91.94	96.65	0.48
5:3	94.03	93.30	97.27	0.55
5.5:2.5	94.52	93.37	97.42	0.49
6:2	94.51	94.50	97.93	0.58
7:1	94.85	94.44	97.17	0.66

**Table 4:** The accuracy (%) table comparison with two basic classifiers.

Method	Accuracy	Std. Deviation
WPD + NN	95.62	0.29
FFT + SAE	94.14	0.34
Fusion module	97.93	0.58

**Table 5:** The accuracy (%) table comparison with other methods.

Method	Feature	Testing Accuracy
SVM [5]	Time Domain	68.67
BP [4]	Time Domain	74.58
DFF [24]	CNN	90.25
ISSVM [23]	WPD	92.25
RF [7]	Time Domain	93.58
Fusion model	/	97.93

## Data Availability

The data presented in this study are available on request from the corresponding author.

## Appendix A

**Table A1:** GJB150.16A-2009 C.7 Vibration Environment Inflection.

Hz	g^2^/Hz	Hz	g^2^/Hz
5	0.0451	104	0.0033
6	0.0303	107	0.0044
7	0.0761	111	0.0032
13	0.0127	147	0.0029
15	0.0327	161	0.0052
16	0.0134	175	0.0022
21	0.0120	233	0.0013
23	0.0261	257	0.0027
25	0.0090	314	0.0016
26	0.0090	333	0.0053
30	0.0137	339	0.0009
34	0.0053	382	0.0017
36	0.0079	406	0.0008
46	0.0039	482	0.0019
50	0.0067	500	0.0007
55	0.0042		
1.29 grms			

## Appendix B

**Table A2:** The time required for the different sections of the fault diagnosis testing period.

Section	Time to First Reuse (ms)	Time to Normal Use (ms)
WPD	70.29	70.29
FFT	41.94	41.94
SAE	15.02	1.01
NN	6.96	0.99
Fusion model	6.98	0.99
Total	141.19	115.22
Time offset	1000.00	
